# Accounting for Population Structure and Phenotypes From Relatives in Association Mapping for Farm Animals: A Simulation Study

**DOI:** 10.3389/fgene.2021.642065

**Published:** 2021-04-29

**Authors:** Enrico Mancin, Daniela Lourenco, Matias Bermann, Roberto Mantovani, Ignacy Misztal

**Affiliations:** ^1^Department of Agronomy, Food, Natural Resources, Animals and Environment, University of Padova, Padua, Italy; ^2^Department of Animal and Dairy Science, University of Georgia, Athens, GA, United States

**Keywords:** genome-wide association, genetic relatedness, single-step genomic best linear unbiased prediction, spurious associations, mixed models, single step genome-wide association study

## Abstract

Population structure or genetic relatedness should be considered in genome association studies to avoid spurious association. The most used methods for genome-wide association studies (GWAS) account for population structure but are limited to genotyped individuals with phenotypes. Single-step GWAS (ssGWAS) can use phenotypes from non-genotyped relatives; however, its ability to account for population structure has not been explored. Here we investigate the equivalence among ssGWAS, efficient mixed-model association expedited (EMMAX), and genomic best linear unbiased prediction GWAS (GBLUP-GWAS), and how they differ from the single-SNP analysis without correction for population structure (SSA-NoCor). We used simulated, structured populations that mimicked fish, beef cattle, and dairy cattle populations with 1040, 5525, and 1,400 genotyped individuals, respectively. Larger populations were also simulated that had up to 10-fold more genotyped animals. The genomes were composed by 29 chromosomes, each harboring one QTN, and the number of simulated SNPs was 35,000 for the fish and 65,000 for the beef and dairy cattle populations. Males and females were genotyped in the fish and beef cattle populations, whereas only males had genotypes in the dairy population. Phenotypes for a trait with heritability varying from 0.25 to 0.35 were available in both sexes for the fish population, but only for females in the beef and dairy cattle populations. In the latter, phenotypes of daughters were projected into genotyped sires (i.e., deregressed proofs) before applying EMMAX and SSA-NoCor. Although SSA-NoCor had the largest number of true positive SNPs among the four methods, the number of false negatives was two–fivefold that of true positives. GBLUP-GWAS and EMMAX had a similar number of true positives, which was slightly smaller than in ssGWAS, although the difference was not significant. Additionally, no significant differences were observed when deregressed proofs were used as pseudo-phenotypes in EMMAX compared to daughter phenotypes in ssGWAS for the dairy cattle population. Single-step GWAS accounts for population structure and is a straightforward method for association analysis when only a fraction of the population is genotyped and/or when phenotypes are available on non-genotyped relatives.

## Introduction

Genome-wide association (GWA) aims to identify regions in the genome that are related to diseases or traits of interest (Begum et al., 2012). The method is most often based on statistical tests to determine if a single nucleotide polymorphism (SNP) is statistically associated with the trait, at a given probability value (*p*-value). If the association is significant, the interrogated SNP may be in high linkage disequilibrium (LD) with a causative variant, or the SNP itself may be a common variant that has a large effect on the trait, although having one or a few causative variants and validating them can be difficult (Kennedy et al., 1992). In fact, results from GWA study (GWAS) have confirmed that most of the complex traits in humans (Yang et al., 2011), animals (de Oliveira Silva et al., 2017), and plants (Bian and Holland, 2017) are polygenic. Even in such a case, the GWAS still fulfills the primary goal of helping to better understand the biology of a trait.

The first GWAS was developed to understand the biology of human diseases aiming the prevention (Burton et al., 2007). Although a couple of studies were published a few years before, the study from 2007 is considered the landmark of GWAS because it resulted from a well-designed, large-scale study (Visscher et al., 2012). After that, GWAS was also adopted in livestock and plants. The very first studies were based on single-SNP analysis where each SNP is tested independently (Balding, 2006). However, this approach assumes SNPs are identically and independently distributed, which is only true when a population is comprised of unrelated individuals (Risch and Merikangas, 1996). As populations contain related individuals, not considering population structure or genetic relatedness in GWAS can result in spurious associations (Sul et al., 2018) To resolve the problem with population structure, the use of principal components (PC) to model relationships have been suggested (Price et al., 2006). Still, the level of confounding in GWAS was considerable when 100 PC were fit into the model or when highly related individuals were removed from a human population (Sul et al., 2018).

A well-known approach among animal breeders, the mixed linear models (Henderson, 1975), was then adopted for human GWAS showing to be a reasonable approach to take population structure into account (Kang et al., 2008, 2010). In this method, known as efficient mixed-model association expedited (EMMAX), one SNP is fit in the model as a fixed covariate and, at the same time, a relationship matrix corrects for population structure. However, EMMAX-based methods consider only genotyped individuals with phenotypes. However, only a fraction of individuals in a population are genotyped, particularly in livestock and aquaculture. Because of that, the original mixed linear models were extended to account for genotyped and non-genotyped individuals in prediction analysis (Aguilar et al., 2010; Christensen and Lund, 2010). This method is called single-step genomic best linear unbiased prediction (ssGBLUP) and is widely adopted for genomic predictions in livestock (Legarra et al., 2014; Misztal et al., 2020) and plants (Cappa et al., 2019), and was recently applied to predict polygenic risk score in humans (Truong et al., 2020). The popularity of ssGBLUP is due to the added value of phenotypes for relatives that are not genotyped, and the simplicity when combining information from genotyped and non-genotyped individuals (Legarra et al., 2014).

The usefulness of ssGBLUP to GWAS in a procedure called single-step GWAS (ssGWAS) was subsequently extended (Wang et al., 2012). In this method, SNP effects and variance explained by SNPs are computed simultaneously for all SNPs while accounting pedigree and genomic relationships in addition to all phenotypes available. However, no statistical significance test was available under the ssGWAS framework. Later it has been shown that the statistical test used in EMMAX has a mathematical equivalent that can be used in GBLUP-based methods (Gualdrón Duarte et al., 2014; Bernal Rubio et al., 2016) even though SNPs are considered fixed in the former and random in the latter. This equivalent statistical test was then implemented in ssGWAS (Aguilar et al., 2019) so that *p*-values are computed based on prediction error variance of SNP effects. Because of the mathematical equivalence, results from ssGWAS are expected to be similar to the ones from EMMAX. Here we use different simulated, structured populations (i.e., beef cattle, dairy cattle, and fish) to investigate the equivalence among EMMAX, ssGWAS, and GBLUP-GWAS, and how they differ from single-SNP analysis. We also evaluate whether the population structure is fully considered by the mixed linear models, and when ssGWAS should be the method of choice for association studies in related populations. We demonstrate ssGWAS performs similarly to EMMAX and GBLUP-GWAS when genotyped animals have their own phenotypes or when only progeny phenotypes are available, so deregressed proofs have to be used for EMMAX and GBLUP-GWAS.

## Materials and Methods

### Methods and Computations

#### Single-SNP Analysis Without Correction for Population Structure (SSA-NoCor)

To estimate allele substitution effect of the i^*th*^ SNP with SSA-NoCor, the following model was used:

(1) $$y=1\mu +x_{i}g_{i}+e$$

where *y* is the vector of phenotypes, μ is the mean, *x*_*i*_ is a vector that contains the genotype for the *i*^*th*^ SNP for each animal, *g*_*i*_ is the *i*^*th*^ allele substitution effect, considered as fixed, and $e∼N\left(0,I\sigma_{e}^{2}\right)$ is the residual. The estimate of $g_{\text{i}}\left({\hat{g}}_{\text{i}}\right)$ and its variance $\left(\text{Var}\left({\hat{g}}_{\text{i}}\right)\right)$ were obtained by least squares.

#### Single-SNP Analysis With Correction for Population Structure Using a Genomic Relationship Matrix (EMMAX)

For the EMMAX method, the estimated allele substitution effect and its variance were obtained from the BLUE of the following linear mixed model:

(2) $$y=1\mu +x_{\text{i}}g_{\text{i}}+\text{Za}+e$$

where *Z* is a design matrix, $a∼N\left(0,G\sigma_{\text{a}}^{2}\right)$ is the vector of breeding values (i.e., animal effect), *G* is the genomic relationship matrix, and the rest of the components were previously defined. The *G* matrix was calculated as first method following literature (Zhou and Stephens, 2012):

(3) $$G=\frac{1}{k}\sum_{i=1}^{k}\left(x_{\text{i}}-1_{\text{n}}{\bar{\text{x}}}_{\text{i}}\right){\left(x_{\text{i}}-1_{n}{\bar{\text{x}}}_{i}\right)}^{T}$$

where *x*_*i*_ stands for i^th^ SNP locus column, ${\bar{\text{x}}}_{\text{i}}$ represented the marker sample mean of *i*^*th*^ locus, *n* and *k* are the number of genotyped animals and SNPs.

#### GBLUP Association (GBLUP-GWAS)

For the GBLUP-GWAS, the vector of estimated allele substitution effects $\hat{g}$ was obtained from a linear transformation of the BLUP of *a* under a GBLUP model:

(4) y=1μ+Za+e

of which the mixed model equations can be represented by:

(5) $$\left[\begin{array}{cc} 1^{{}^{\prime }}1 & 1^{{}^{\prime }}Z\\ {\text{Z}}^{{}^{\prime }}1 & {\text{Z}}^{{}^{\prime }}Z+{\text{G}}^{-1}\frac{\sigma_{\text{e}}^{2}}{\sigma_{\text{a}}^{2}} \end{array}\right]\left[\begin{array}{c} \hat{\mu }\\ \hat{a} \end{array}\right]=\left[\begin{array}{c} 1^{{}^{\prime }}y\\ Z^{{}^{\prime }}y \end{array}\right]$$

In this method, *G*_0_ was estimated using the first method of VanRaden (2008):

(6) $$G_{0}=\frac{{\text{MM}}^{\prime }}{2\sum p_{\text{i}}\left(1-p_{\text{i}}\right)}$$

where *M* is a matrix of SNP content centered by twice the current allele frequencies, and *p*_*i*_ is the allele frequency for the i^th^ SNP. Additionally, to avoid singularity problems, the final *G* was computed as

(7) $$G=\lambda G_{0}+\beta I$$

with λ = 0.95 and β = 0.05.

Afterward, the vector of allele substitution effects ($\hat{g}$) was calculated for all SNPs simultaneously (Wang et al., 2012):

(8) $$\hat{g}=\lambda \frac{1}{2\sum pq}M^{\prime }G^{-1}\hat{a}$$

with *q* = 1−*p*.

The variance of SNP effects, which is needed to compute *p*-values when SNPs are considered random was calculated following Gualdrón Duarte et al. (2014):

(9) $$Var\left(\hat{g}\right)=\lambda \frac{1}{2\sum pq}Z^{\prime }G^{-1}\left(G{\hat{\sigma }}_{a}^{2}-C^{22}\right)G^{-1}Z\lambda \frac{1}{2\sum pq}$$

where *C*^22^ is the block of the inverse of the MME corresponding to the animal effect. The *p*-value for each SNP effect was then computed with the formula (Gualdrón Duarte et al., 2014):

(10) $$p-value_{\text{i}}=2\left(1-\Phi \left(\left|\frac{{\hat{g}}_{\text{i}}}{sd\left({\hat{g}}_{\text{i}}\right)}\right|\right)\right)$$

where $sd\left({\hat{g}}_{\text{i}}\right)$ is the standard error of the SNP effect or simply $sd\left({\hat{g}}_{\text{i}}\right)=\sqrt{Var\left({\hat{g}}_{\text{i}}\right)}$; Φ(⋅) is the cumulative density function (CDF) of the standard normal distribution. For a justification of using $sd\left({\hat{g}}_{\text{i}}\right)=\sqrt{Var\left({\hat{g}}_{\text{i}}\right)}$ in the denominator instead of $\sqrt{Var\left(g_{\text{i}}\right)-Var\left({\hat{g}}_{\text{i}}\right)}$ (Gualdrón Duarte et al., 2014; Bernal Rubio et al., 2016).

#### Single-Step GBLUP Association (ssGWAS)

This method differs from GBLUP-GWAS in the sense that all animals in the pedigree can be used, not only genotyped animals with phenotypes. Therefore, *G*^−1^ is replaced by *H*^−1^ in (5), and the latter combines pedigree and genomic relationships (Aguilar et al., 2010):

(11) $$H^{-1}=A^{-1}+\left[\begin{array}{cc} 0 & 0\\ 0 & G^{-1}-A_{22}^{-1} \end{array}\right]$$

where *A*^−1^ and $A_{22}^{-1}$ are the inverses of the pedigree relationship matrix for all animals and only genotyped animals, respectively. Pedigree and genomic relationships have different genetic base because allele frequencies from the current genotyped population are used to center *G*. Therefore, *G* in ssGWAS is adjusted so the average diagonal and off-diagonal matches the averages of *A*_*22*_. Because of this adjustment, (8) and (9) were modified to:

(12) $$\hat{g}=\lambda \delta \frac{1}{2\sum pq}M^{\prime }G^{-1}{\hat{a}}_{22}$$

and

(13) $$Var\left(\hat{g}\right)=\lambda \delta \frac{1}{2\sum pq}Z^{\prime }G^{-1}\left(G{\hat{\sigma }}_{\text{a}}^{2}-C^{22}\right)G^{-1}Z\lambda \delta \frac{1}{2\sum pq}$$

where ${\hat{a}}_{22}$ is a vector of genomic estimated breeding values (GEBV) for genotyped animals; δ accounts for the difference in genetic base between the pedigree and genomic relationship matrices, and was calculated as in Vitezica et al. (2011):

(14) $$\delta =1-\frac{0.5}{n^{2}}\left(\sum_{\text{i}}\sum_{\text{j}}A_{22\left(i,j\right)}-\sum_{\text{i}}\sum_{\text{j}}G_{i,j}\right)$$

with *n* the number of genotyped animals. After the modification, *p*-values in ssGWAS were obtained as in (10) as previously suggested (Aguilar et al., 2019).

Note that the dimension of ssGWAS system of equations is greater than the dimension of GBLUP-GWAS because of the inclusion of non-genotyped animals.

#### Computations

The allele substitution effects were estimated with different software: (i) SSA-NoCor solutions were computed with GASTON R-package (Dandine-Roulland and Perdry, 2018), (ii) EMMAX solutions were obtained with GEMMA software (Zhou and Stephens, 2012), (iii) GBLUP-GWAS and (iv) ssGWAS were computed using the BLUPF90 software suite (Misztal et al., 2014b).

### Overview of Data Simulation

**FIGURE 1:**
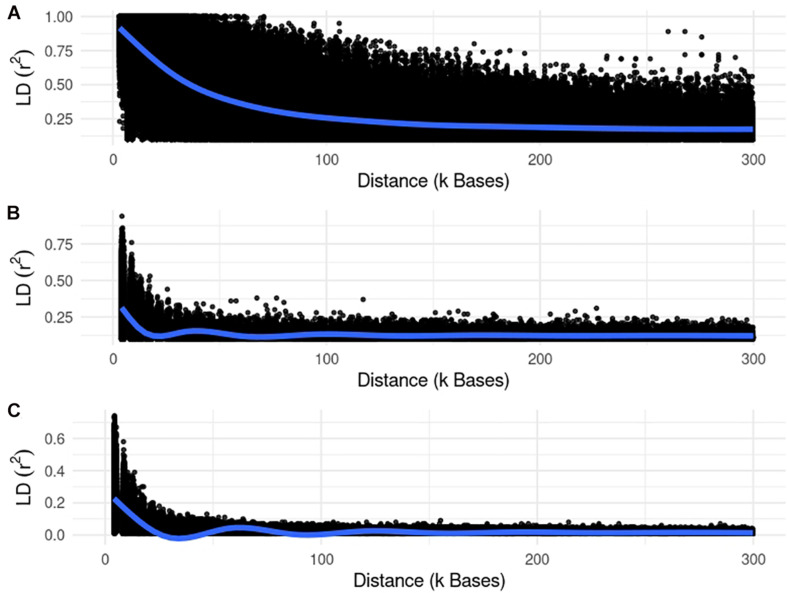
Linkage disequilibrium (r2) decay for the small populations of fish **(A)**, beef **(B)**, and dairy cattle **(C)**.

**FIGURE 2:**
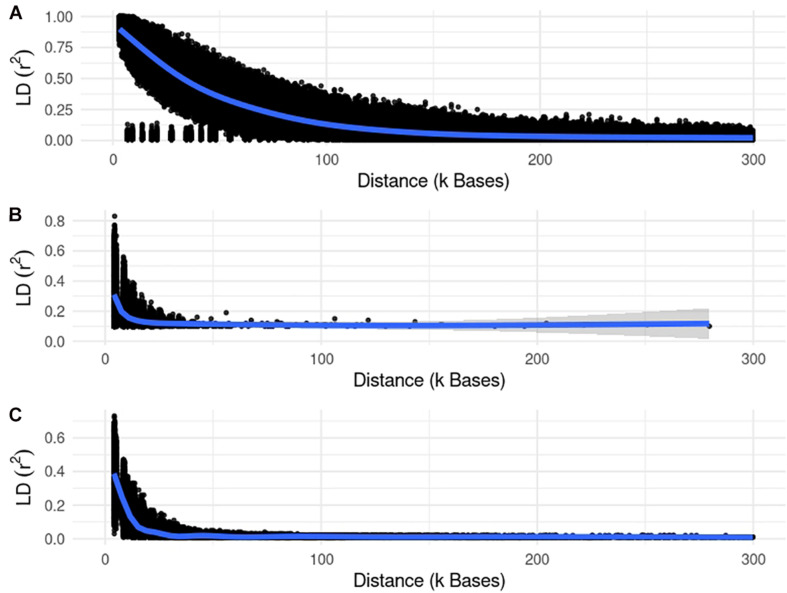
Linkage disequilibrium (r2) decay for the large populations of fish **(A)**, beef **(B)**, and dairy cattle **(C)**.

We used different simulated datasets to investigate the equivalence between EMMAX and ssGWAS, and to explore the usefulness of each method compared to SSA-NoCor and GBLUP-GWAS. Small and large fish, beef cattle, and dairy cattle populations were simulated using QMSim (Sargolzaei and Schenkel, 2009), with five replicates for each. Because we wanted to test the efficiency of the methods in accounting for population structure, strong assumptions were used to generate extreme scenarios. The general parameters for each population such as the number of genotyped animals, effective population size, type of trait, and heritability are reported in Table 1. The linkage disequilibrium generated in each small and large populations are in Figures 1, 2.

**TABLE 1:** General parameters for the simulated populations^1,2^.

**Simulated populations**	**Number of records**	**Population size**	**Number of genotyped animals**	**Number of genotyped animals with records^1^**	**Trait type**	**Heritability**
**Small populations**						
Fish	2040	2040	1040	1040	Both sex	0.25
Beef	5088	10,000	5525	3010	Sex-limited	0.30
Dairy	70,000 (0.38)	140,000	1438	1438	Sex-limited	0.35
**Large populations**						
Fish	20,400	20,400	10,400	10,400	Both sex	0.25
Beef	25,000	50,000	14,250	8985	Sex-limited	0.30
Dairy	250,000 (0.37)	500,000	14,000	14,000	Sex-limited	0.35

*^1^For the dairy population, the records are represented as deregressed proofs. The average reliability of deregressed proofs is reported inside parenthesis. ^2^For beef populations, association studies with SSA-NoCor, EMMAX, and GBLUP-GWAS were performed only with genotypes for the sex that had records.*

#### Fish Population

The historical population began with 5,000 animals and decreased to 3,100 after 200 non-overlapping generations, that were carried out to generate linkage disequilibrium (LD) and mutation-drift equilibrium. The proportion of males in the historical population was 32%. Aiming to mimic the beginning of a fish breeding scheme, a recent population was created by randomly selecting 20 sires and 20 dams. The recent population was subject to random selection, culling, and mating for five non-overlapping generations (Garcia et al., 2018). In every generation, each female had 2 offspring. After the fifth generation, a new line was created by randomly mating 20 males and 20 females from generation 5. For this line, the litter size was set to 100 offspring per dam, and only one generation was created. The effective population size was 40. Phenotypes and pedigree of the animals in the new line, together with their parents, were considered for the association analysis. A larger fish population was simulated that had 10 times more animals (Table 1).

The genome was composed of 29 chromosomes with a length of approximately 100 cM each, 35,000 evenly spaced SNPs, and one QTN per chromosome. Each QTN was placed in the middle of its respective chromosome. Although this number of QTN is not the reality of most of the traits of interest (i.e., complex traits), this assumption was made to facilitates the QTN discovery in the association analysis. Altogether, the QTNs accounted for the total of the genetic variation and their effects were assumed to follow a gamma distribution with shape parameter equal to 0.40. The allele frequencies for SNPs and QTNs in the first historical generation were 0.5, and a recurrent mutation rate of 2.5e-5 per locus per generation was assumed. A single trait with heritability of 0.25 was simulated, and a single phenotype per animal was obtained by adding an overall mean of 1.0, the sum of the QTN effects, and a residual effect.

#### Beef Cattle Population

In this dataset, the historical population began with 1,000 animals and steadily increased to 50,000 after 1,000 generations of random mating. Then, a decrease in number of individuals followed for another 1,000 generations. After 2,000 generations, the historical population was composed by 23,000 animals, of which 3,000 were males. The recent beef cattle population was created by randomly selecting 10,000 dams and 200 sires, allowing them to mate randomly for five discrete generations. Afterward, five groups of 10 sires and 500 dams each were selected based on EBV to create five different lines. With the aim of maximizing the difference between the five lines, selection based on EBV was used in each of them. Finally, 10 sires and 500 dams from each of the five lines were pooled in one single line and underwent random mating for five generations. This process was designed to create an intricate population structure. For the present population, a sex-limited trait was simulated so that only females had a phenotype for a trait with heritability of 0.30. A larger beef cattle population was simulated that had 5 times more animals (Table 1).

Genotypes were simulated for males and females from the last generation of the population and their parents (*n*_*beef*_ = 5525). The parameters to simulate the beef cattle genome were the same as in the fish population except for the number of SNPs, which for the beef cattle population was equal to 65,000.

#### Dairy Cattle Population

The parameters for the simulation of the dairy historical population were the same as those in beef cattle. A total of 1,000 sires and 20,000 were chosen as founders of the recent population. This population was subject to selection based on estimated breeding values (EBV) for 10 generations and mating design based on inbreeding (Sonesson and Meuwissen, 2000). In this simulation, the average Ne was 90. The Ne was calculated as the change in inbreeding (Δ*F*) from one generation to the next using the following formula (Falconer and Mackay, 1996):

(15) $$\Delta F=\frac{F_{\text{n}}-F_{\text{n}-1}}{1-F_{\text{n}-1}}$$

(16) $$Ne=\frac{1}{2\Delta F}$$

where *F*_*n*_ is the inbreeding in the *nth* generation.

All the parameters for the genome simulation were similar to the ones in the beef cattle population. The only difference was the genotyping strategy that included only sires of the seventh generation (*n*_*dairy*_ = 1400). Phenotypes for a trait of heritability 0.35 were available only on females (Table 1). On average, each genotyped sire in this population had 10 daughters with records. A larger dairy cattle population was simulated that had 3.5 times more animals and 10 times more genotyped bulls (Table 1).

### Deregressed Proofs (DRP)

One requirement in association analysis is that individuals should have both genotypes and phenotypes. In some livestock populations, genotypes may be available for males and phenotypes for females (e.g., milk production in dairy cattle). In such a case, DRP are needed as an input for SSA-NoCor, EMMAX, or GBLUP-GWAS. The DRP are projections of female phenotypes into their relatives’ genotypes. Because sex-limited traits were simulated for the beef and dairy cattle populations, DRP were computed for sires in both populations following (VanRaden and Sullivan, 2010; Wiggans et al., 2011).

(17) $$DRP=PA+\frac{EBV-PA}{DE_{\text{prog}}/\left(DE_{\text{prog}}+DE_{\text{PA}}+1\right)}$$

Where *PA* is parent average; $DE_{\text{prog}}=\left[\frac{EBV_{\text{rel}}}{\left(1-EBV_{\text{rel}}\right)}\right]-DE_{\text{PA}}$ and is the daughter equivalent from progeny information; and $DE_{\text{PA}}=\frac{PA_{\text{rel}}}{\left(1-PA_{\text{rel}}\right)}$ is the daughter equivalent from PA. The *EBV*_*rel*_ and *PA*_*rel*_ are reliabilities of parent average and EBV, respectively. All EBV, PA, and reliabilities used in the DRP formula were computed using the BLUPF90 software suite (Misztal et al., 2014b). The DRP were used for the association analysis of dairy cattle datasets under SSA-NoCor, EMMAX, and GBLUP-GWAS. As ssGWAS uses all phenotypes, genotypes, and pedigree information available, it does not rely on DRP.

### Quality Control Prior to the Association Analysis

Quality control of genomic data removed monomorphic SNPs, SNPs with minor allele frequency (MAF) lower than 0.05, and with deviation between observed and expected allele frequencies greater than 0.15. After quality control, and average of 35,000, 58,000, and 58,000 SNPs were kept for the analysis in the fish, beef cattle, and dairy cattle population, respectively.

### Significance and Concordance Tests

A single SNP was considered significantly associated with the considered trait when its *p*-value was smaller than a certain significance level, which was 0.05 with a Bonferroni correction for multiple testing, i.e., $\frac{0.05}{n}$ where *n* is the number of SNPs in the corresponding simulated population. Additionally, true positive (TP) and false positive (FP) rates were computed for each scenario using a window size of ±2 cM, which is equivalent to 20–30 markers (Toosi et al., 2018).

## Results and Discusion

### Population Structure

**FIGURE 3:**
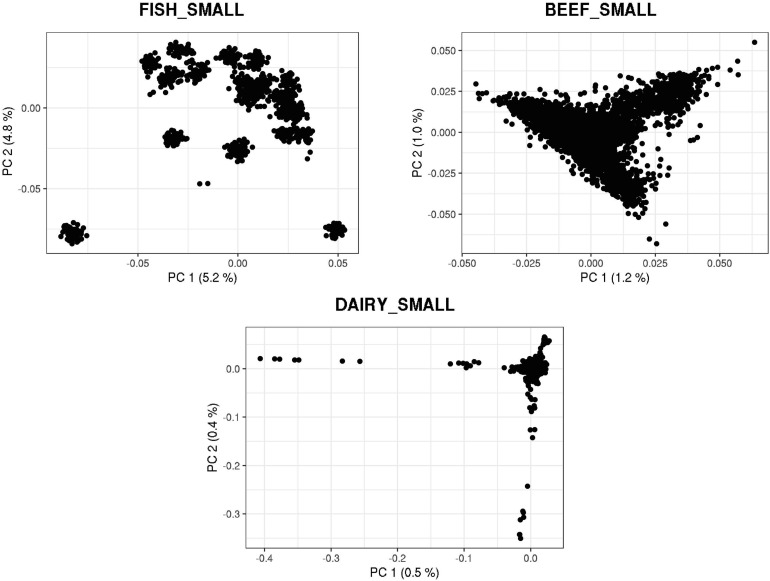
First and second principal components of the genomic relationship matrices for the small populations of fish, beef, and dairy cattle.

**FIGURE 4:**
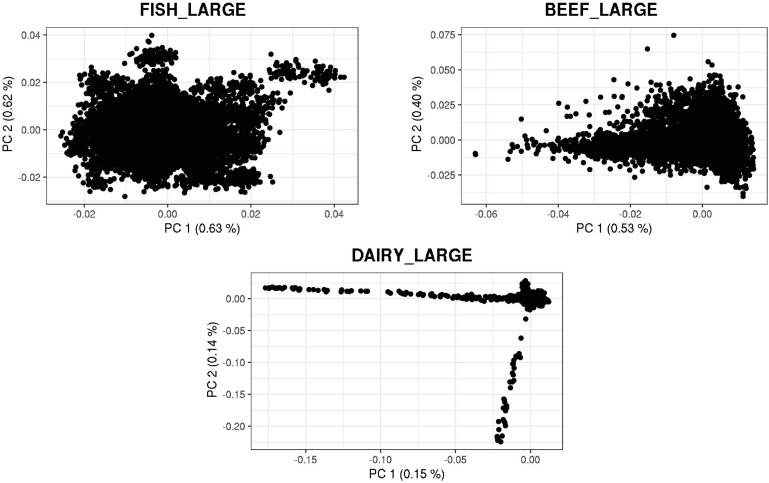
First and second principal components of the genomic relationship matrices for the large populations of fish, beef, and dairy cattle.

Figures 3, 4 show plots with the first (PC1) and second (PC2) principal components of *G* for small and large populations, respectively. PC1 and PC2 represent the two largest sources of variation in the data, and are often used to investigate population structure, which was deemed important in our study. The level of population stratification differed among the simulated populations because of the different selection and mating strategies. For the small populations, distinct family groups (full-sibs) were observed for the fish population, with variable size and impact on the model (extreme clusters farther from PC1 = PC2 = 0); however, the PC scores were of small magnitude. No distinct clustering was detected in the beef cattle population, although a level of variability was observed. Overall, it was not possible to discriminate different groups of individuals but some of them appear more genetically different than others. In fact, animals belonging to five different lines were randomly mated for ten generations, and only genotypes for animals in the 7th generation were retained. Therefore, less genetic distance among animals was created. Still, the largest graphical distance was observed in the dairy cattle population, with a large cluster centered in zero and a few animals genetically distant from the main cluster; however, the amount of variation explained by PC1 and PC2 was small. Possibly, this pattern resulted from non-overlapping generations that created extra genetic distance among some animals. These distant animals were sires that had EBV departing from the population mean. For the large populations, no clustering was observed for the fish and beef populations, whereas the same pattern was observed for dairy cattle in both populations.

### Association Analysis

**FIGURE 5:**
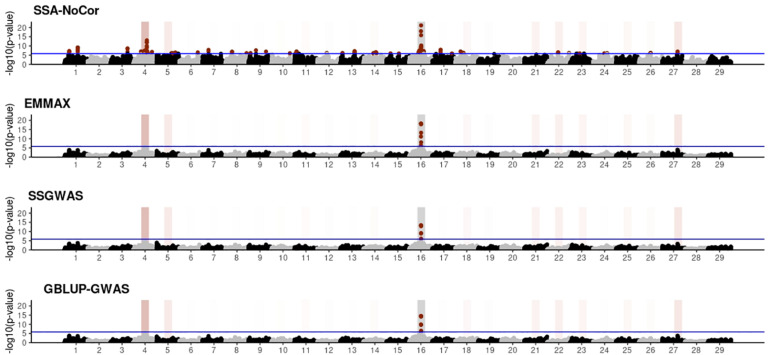
Manhattan plots for the small population of fish using single-SNP analysis without correction for population structure (SSA-NoCor), efficient mixed-model association expedited (EMMAX), single-step GWAS (ssGWAS), and genomic best linear unbiased prediction GWAS (GBLUP-GWAS). Significant SNPs are indicated in red, whereas vertical bars indicate the position of the simulated quantitative trait nucleotide (QTN). The darker the vertical bar, the stronger QTN effect. The blue horizontal line corresponds to the rejection threshold based on a significance level of 0.05 with a Bonferroni correction for multiple testing.

**FIGURE 6:**
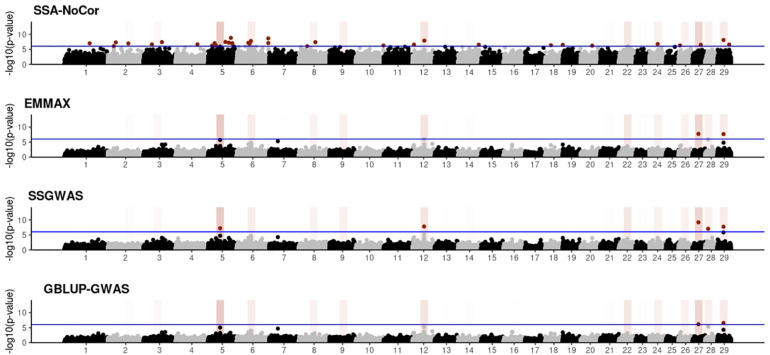
Manhattan plots for the small population of beef cattle using single-SNP analysis without correction for population structure (SSA-NoCor), efficient mixed-model association expedited (EMMAX), single-step GWAS (ssGWAS), and genomic best linear unbiased prediction GWAS (GBLUP-GWAS). Significant SNPs are indicated in red, whereas vertical bars indicate the position of the simulated quantitative trait nucleotide (QTN). The darker the vertical bar, the stronger QTN effect. The blue horizontal line corresponds to the rejection threshold based on a significance level of 0.05 with a Bonferroni correction for multiple testing.

**FIGURE 7:**
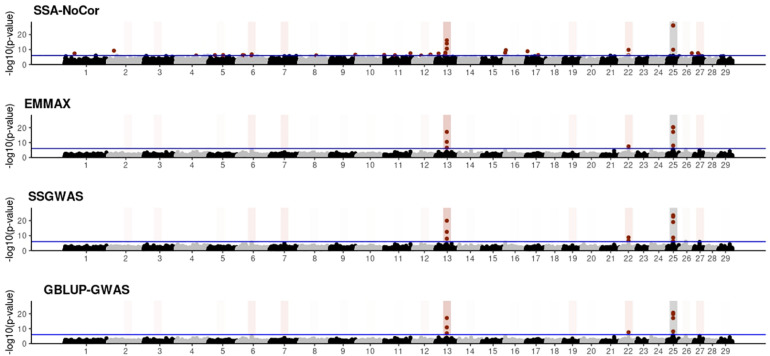
Manhattan plots for the small population of dairy cattle when sires had an average of ten daughters. The association methods used were single-SNP analysis without correction for population structure (SSA-NoCor), efficient mixed-model association expedited (EMMAX), single-step GWAS (ssGWAS), and genomic best linear unbiased prediction GWAS (GBLUP-GWAS). Significant SNPs are indicated in red, whereas vertical bars indicate the position of the simulated quantitative trait nucleotide (QTN). The darker the vertical bar, the stronger QTN effect. The blue horizontal line corresponds to the rejection threshold based on a significance level of 0.05 with a Bonferroni correction for multiple testing.

**FIGURE 8:**
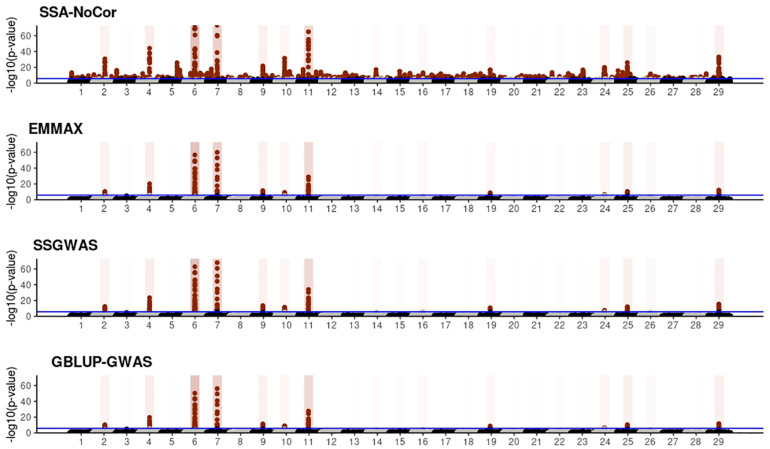
Manhattan plots for the large population of fish using single-SNP analysis without correction for population structure (SSA-NoCor), efficient mixed-model association expedited (EMMAX), single-step GWAS (ssGWAS), and genomic best linear unbiased prediction GWAS (GBLUP-GWAS). Significant SNPs are indicated in red, whereas vertical bars indicate the position of the simulated quantitative trait nucleotide (QTN). The darker the vertical bar, the stronger QTN effect. The blue horizontal line corresponds to the rejection threshold based on a significance level of 0.05 with a Bonferroni correction for multiple testing.

**FIGURE 9:**
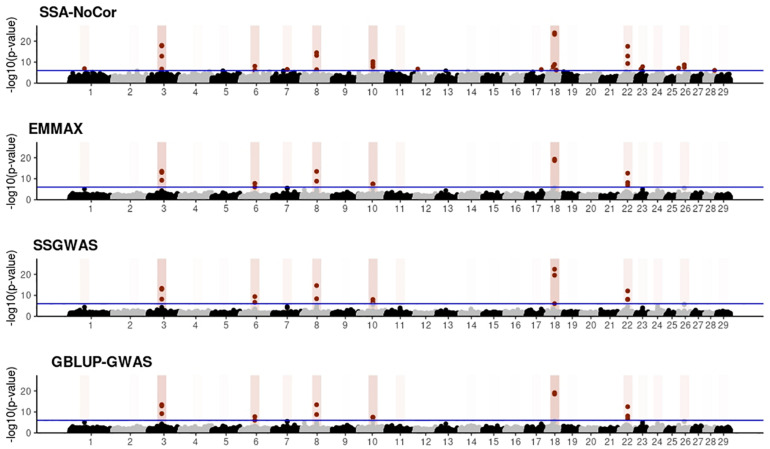
Manhattan plots for the large population of beef cattle using single-SNP analysis without correction for population structure (SSA-NoCor), efficient mixed-model association expedited (EMMAX), single-step GWAS (ssGWAS), and genomic best linear unbiased prediction GWAS (GBLUP-GWAS). Significant SNPs are indicated in red, whereas vertical bars indicate the position of the simulated quantitative trait nucleotide (QTN). The darker the vertical bar, the stronger QTN effect. The blue horizontal line corresponds to the rejection threshold based on a significance level of 0.05 with a Bonferroni correction for multiple testing.

**FIGURE 10:**
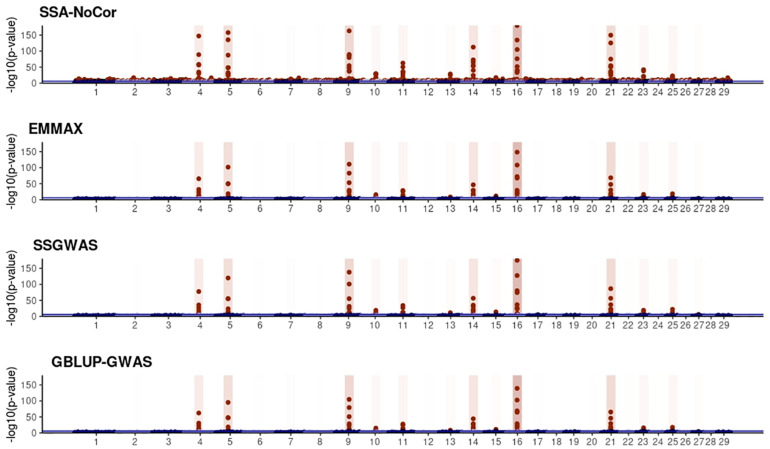
Manhattan plots for the large population of dairy cattle when sires had an average of ten daughters. The association methods used were single-SNP analysis without correction for population structure (SSA-NoCor), efficient mixed-model association expedited (EMMAX), single-step GWAS (ssGWAS), and genomic best linear unbiased prediction GWAS (GBLUP-GWAS). Significant SNPs are indicated in red, whereas vertical bars indicate the position of the simulated quantitative trait nucleotide (QTN). The darker the vertical bar, the stronger QTN effect. The blue horizontal line corresponds to the rejection threshold based on a significance level of 0.05 with a Bonferroni correction for multiple testing.

Manhattan plots with *p*-values for the fish, beef cattle, and dairy cattle populations using SSA-NoCor, EMMAX, and ssGWAS, and GBLUP-GWAS are in Figures 5–10. Although one QTN was simulated in each chromosome, the signals were not equally strong because of the assumption of a Gamma distribution, and the selection that the populations underwent. Overall, selection caused fixation for 5–6% of the QTN. To better access the information in the Manhattan plots, the average number of true and false positive SNPs were computed and placed in Table 2. Although the number of TP and FP differed among EMMAX, ssGWAS, and GBLUP-GWAS, the differences were not statistically significant (*p*-value > 0.05).

**TABLE 2:** Average (SD) number of true positive (TP) and false positive (FP) SNPs for all the simulated populations^1^.

Population	Association	SSA_NoCor	EMMAX	ssGWAS	GBLUP-GWAS
**Small populations**					
Fish	TP	85.20(0.47)^a^	20.00(0.31)^b^	20.60(0.35)^b^	20.00(0.31)^b^
	FP	253.6(2.50)^a^	3.80(1.01)^b^	5.20(1.18)^b^	2.80(0.96)^b^
Beef	TP	15.80(0.68)^a^	5.60(0.73)^a^	7.60(0.50)^a^	5.60(0.73)^a^
	FP	68.60 (0.78)	0.00	0.20 (2.24)	0.00
Dairy	TP	13.40(0.20)^a^	8.60(0.60)^a^	12.60(0.12)^a^	9.00(0.55)^a^
	FP	24.20 (0.32)	0.00	0.20 (2.24)	0.20 (2.24)
**Large populations**					
Fish	TP	321.40(72.52)^a^	106.20(25.41)^b^	133.80(37.93)^b^	104.20(25.33)^b^
	FP	1068 (241.67)	0.00	0.00	0.00
Beef	TP	24.60(3.29)^a^	17.20(5.54)^a^	21.00(5.34)^a^	17.00(5.48)^a^
	FP	4.40(2.19)^a^	0.40(0.55)^b^	0.20(0.45)^b^	0.40(0.55)^b^
Dairy	TP	151.60(5.98)^a^	55.20(3.7)^b^	61.60(8.65)^b^	52.40(5.94)^b^
	FP	853.80 (39.32)	0.00	0.00	0.00

*^1^Statistical differences were not calculated when the SD of at least one method within a group was null. The superscripts within a row, group the results that did not differ significantly.*

For all the simulated populations, the greatest number of false positive SNPs was observed for SSA-NoCor. These results agree with those from previous studies (e.g., Yang et al., 2014), which showed that the number of false positives drastically decreased when correcting for population structure. For the small populations, the number of false positive SNPs in SSA-NoCor for the dairy dataset was the smallest one compared to the other simulated populations, whereas for the large populations, the smallest number of false positive SNPs occurred in the beef population. In both cases, the fish population had the greatest number of false positive signals. Since this population had a strong structure (e.g., several separate clusters), it can be concluded that the population structure is related to the number of false positive signals captured by SSA-NoCor. False positive associations capture SNPs that relate to the genetic differences between sub-populations and also with the trait considered (Sul et al., 2018). These spurious signals can also be interpreted as a wrong prior assumption of marker effects in the SSA-NoCor model. In such a model, markers are considered independently distributed, which implicitly means linkage disequilibrium among SNPs is neglected (Finno et al., 2014; Sul et al., 2018). The effect of population structure is even more evident when small sample size and high-density panels are used in association analyses (Finno et al., 2014). Furthermore, when traits are polygenic or have low heritability, signals deriving from population structure can completely override those deriving from true QTNs (Atwell et al., 2010; Toosi et al., 2018).

In terms of true positives, two situations were observed. First, SSA-NoCor detected significantly more true positives than the other methods in the fish and large dairy cattle simulated populations. However, the number of false positives was sometimes threefold greater than that of true positives. The identification of false and true positives is straightforward in simulated data but not in real data. Second, no significant differences were observed among methods for the beef and the small dairy population. Based on the results from the dairy population, it can be concluded that the use of DRP for bulls in EMMAX and GBLUP-GWAS, compared to the raw phenotypes in ssGWAS, did not promote a loss in GWA resolution. The loss in the ability to correctly detect QTNs when using DRP is expected in complex models when the estimation of fixed and random effects is not very accurate. According to Aguilar et al. (2019), information can be lost in the deregression process, which may result in spurious signals in GWA. Although DRP were used in the dairy population, no significant differences in TP and FP were observed between EMMAX and ssGWAS because the model was simple and included only a general mean as fixed effect and the additive genetic as random.

**FIGURE 11:**
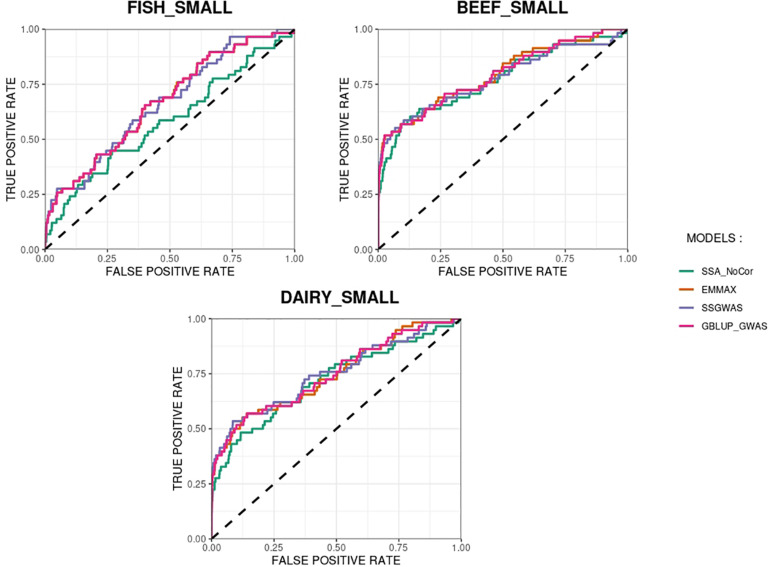
Receiver operating characteristic (ROC) curves for GWAS results for the small populations of fish, beef cattle, dairy cattle with ten daughters per sire. The association methods used were single-SNP analysis without correction for population structure (SSA-NoCor), efficient mixed-model association expedited (EMMAX), single-step GWAS (ssGWAS), and genomic best linear unbiased prediction GWAS (GBLUP-GWAS). The dashed line has slope equal to one and null intercept.

**FIGURE 12:**
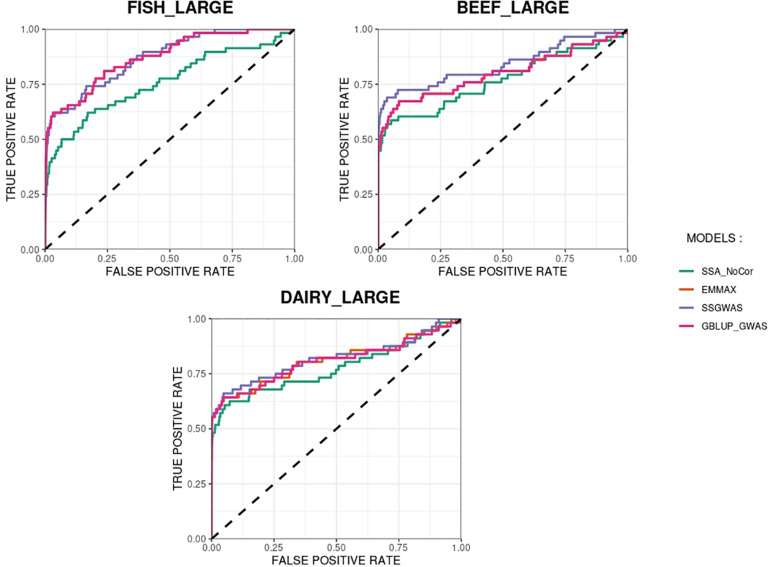
Receiver operating characteristic (ROC) curves for GWAS results for the large populations of fish, beef cattle, dairy cattle with ten daughters per sire. The association methods used were single-SNP analysis without correction for population structure (SSA-NoCor), efficient mixed-model association expedited (EMMAX), single-step GWAS (ssGWAS), and genomic best linear unbiased prediction GWAS (GBLUP-GWAS). The dashed line has slope equal to one and null intercept.

The methods in our study were used as binary classifiers when trying to identify true and false positives. The quality of a binary classifier can be evaluated from the degree of randomness of the decisions of that classifier. A perfect classifier is not random, whereas the worst classifier would determine whether a signal is true or false with a probability equal to 0.5 (Agresti, 2013). To compare the methods in our study as binary classifiers, Receiver Operating Characteristic (ROC) curves were provided for each simulated population in Figures 11, 12. Among the plots, it can be observed that the curve corresponding to SSA-NoCor is the lowest one. Therefore, as a binary classifier, SSA-NoCor performs worse than the rest of the methods. The classifier ability of the models improved in the large populations, but SSA-NoCor still had poorer performance compared to other methods. The fact large data improves the resolution of GWAS is well documented in the literature.

Overall, we observed the size of the populations (e.g., small and large) did not change the outcome of our study, and we confirmed, using simulated populations with intricate structure, that EMMAX, GBLUP-GWAS, and ssGBLUP account for population structure. The equivalence between *p*-values obtained in EMMAX and GBLUP-GWAS has been analytically demonstrated (Bernal Rubio et al., 2016), although the former considers SNPs as fixed effects and the latter as random. Lu et al. (2018) extended this idea to single-step and implemented it with the addition of *p*-values for ssGWAS in the BLUPF90 software suite (Misztal et al., 2014b; Aguilar et al., 2019). This methodology was successfully applied to a beef cattle population with almost 2 million animals in the pedigree, 1 million birth weight records, and a little over 1,400 genotyped sires (Aguilar et al., 2019). In our study, we confirmed that ssGWAS can account for population structure as EMMAX or GBLUP-GWAS.

Recently, single-step was applied for predicting polygenic risk score in humans using phenotypes from related individuals that were not genotyped (Truong et al., 2020). In this study, authors observed an increase in prediction accuracy when raw phenotypes of non-genotyped relatives were included in the model, which is only possible with single-step method. As the number of genotyped individuals in (Legarra et al., 2009; Truong et al., 2020) was 288k, the authors complained about the computing cost of single-step, which is mainly due to the inverse of *G*. An efficient algorithm to compute *G*^−1^ without having to directly invert *G* –the Algorithm for Proven and Young (APY)– is also available (Misztal et al., 2014a). With the APY algorithm, animals are designated as core or non-core, and recursions are done on core animals, whereas predictions for non-core animals are functions of the information for core animals. This is possible because of the assumption that core animals carry all the information about the independent chromosome segments segregating in the population (Misztal, 2016). In addition, it was found (Pocrnic et al., 2016) that the number of largest eigenvalues explaining 98% of the variance in *G* approaches the number of independent chromosome segments (Stam, 1980) and can be used as the number of core animals in APY. This algorithm enables the computation of genomic predictions for millions of genotyped individuals with much less memory usage and computing time. Indeed, a successful computation of genomic predictions for 13.5 million animals in the pedigree, of which 2.3 million were genotyped, using the BLUPF90 software suite has recently been shown to be feasible (Tsuruta et al., 2021). Although the computation of genomic predictions (GEBV), SNP effects, and variance explained by SNPs can be done efficiently with APY in ssGBLUP, the same does not apply to the computation of *p*-values in ssGWAS. This is because the formula for *p*-values (10) relies on the standard error of SNP effects (i.e., square root of prediction error variance), which is currently obtained based on the prediction error variance of GEBV. The latter requires the inverse of the left-hand-side of the single-step mixed model equations, and the computation of inverses of large matrices is extremely expensive. Therefore, the use of ssGWAS may be limited to samples of about 20k genotyped individuals, given the number of total animals in the pedigree is less than 500k. Approximating the prediction error variance of GEBV or SNPs directly may be a way to overcome this limitation, and research on the issue is currently undergoing.

## Conclusion

Genome-wide association studies in related populations require the correction for population structure to avoid false positive statistical associations between SNPs and trait phenotypes. Several classes of mixed linear models as EMMAX, GBLUP-GWAS, and ssGWAS can take care of this issue by fitting a random effect whose covariance matrix is proportional to a relationship matrix. We demonstrate the three methods did not significantly differ across association studies in several simulated populations, regardless if deregressed proofs or phenotypes from non-genotyped animals are used in the statistical analysis. Further studies are needed to investigate the repeatability of those results in real populations under complex models. Single-step GWAS accounts for population structure as EMMAX or GBLUP-GWAS and allows for the inclusion of phenotypes from non-genotyped relatives.

## Data Availability Statement

The original contributions generated for this study are included in the article/supplementary material, further inquiries can be directed to the corresponding author.

## Author Contributions

EM performed the analysis and simulation and writing. DL supervised all the analysis, design the global structure of the simulations, and writing. MB revised the manuscript and helpful comments and idea. RM revised the manuscript. IM head of the project. All authors contributed to the article and approved the submitted version.

## Conflict of Interest

The authors declare that the research was conducted in the absence of any commercial or financial relationships that could be construed as a potential conflict of interest.
